# The PSRP2/4 Proteins Promote Viral Infection by Interacting with the VPg Protein of TuMV

**DOI:** 10.3390/plants14203211

**Published:** 2025-10-19

**Authors:** Shanwu Lyu, Wenjun Lu, Changwei Zhang, Wenlong Wang, Mengguo Yuan, Liu E, Tingting Liu, Shulin Deng

**Affiliations:** 1 Key Laboratory of National Forestry and Grassland Administration on Plant Conservation and Utilization in Southern China, Guangdong Provincial Key Laboratory of Applied Botany, South China Botanical Garden, Chinese Academy of Sciences, Guangzhou 510650, China; t2019113@njau.edu.cn (S.L.); liutingting231@mails.ucas.ac.cn (T.L.); 2 National Key Laboratory of Crop Genetics & Germplasm Enhancement and Utilization, College of Horticulture, Nanjing Agricultural University, Nanjing 210095, China; 2023104067@stu.njau.edu.cn (W.L.); changweizh@njau.edu.cn (C.Z.); 2023803177@stu.njau.edu.cn (W.W.); 2020104077@stu.njau.edu.cn (M.Y.); 2021104078@stu.njau.edu.cn (L.E.)

**Keywords:** plastid-specific ribosomal proteins, *BcPSRP2/4*, host factor, turnip mosaic virus, non-heading Chinese cabbage

## Abstract

Chloroplasts, which are essential for plant defense and phytohormone signaling, contain ribosomal proteins that play key roles in viral infection processes. Plastid-specific ribosomal proteins (PSRPs), unique to chloroplasts, remain unexplored in their mechanistic roles during plant-virus interactions. In this study, we identified two PSRPs from non-heading Chinese cabbage (*Brassica campestris* ssp. *chinensis*) as interacting with turnip mosaic virus (TuMV, *Potyvirus rapae*). Subcellular localization revealed that BcPSRP2/4 is targeted to chloroplasts, while BiFC, Y2H, and LCAs confirmed their interaction with TuMV VPg (virus protein, genome-linked). Intriguingly, VPg altered the subcellular localization of BcPSRP2/4, suggesting an important role for BcPSRP2/4 in TuMV infection. Strikingly, overexpression of *BcPSRP2/4* enhanced TuMV cell-to-cell movement, while *psrp2* knockdown mutants in Arabidopsis exhibited a significant reduction in viral accumulation, highlighting their proviral roles. Furthermore, virus-induced gene silencing (VIGS)-mediated suppression of *BcPSRP2/4* in non-heading Chinese cabbage resulted in milder symptoms upon TuMV infection without compromising plant growth: a distinct advantage over conventional resistance genes that incur fitness costs. These findings highlight *PSRP2/4* as pivotal molecular hinges in chloroplast-virus interplay, offering novel targets for engineering sustainable antiviral strategies in cruciferous crops.

## 1. Introduction

Turnip mosaic virus (TuMV, *Potyvirus rapae*) is a member of the genus *Potyvirus* within the family *Potyviridae* and was first identified in *Brassica rapa* in the United States in 1921 [1]. It is widely distributed globally, causing significant damage to crops, particularly in Asia, North America, and Europe [2]. This virus has a broad host range, infecting at least 318 plant species across 43 families, including Cruciferae, Compositae, Chenopodiaceae, Leguminosae, and Caryophyllaceae, as well as some monocots [3]. TuMV poses a particularly severe threat to Brassica crops, such as non-heading Chinese cabbage, radish, broccoli, and mustard, making it a significant concern for agricultural productivity worldwide. Controlling TuMV is complicated by its non-persistent spread via 89 aphid species [4,5] and climate-driven instability of temperature-sensitive monogenic resistance under rising temperatures [6]. As a positive-sense single-stranded RNA virus, TuMV relies heavily on host cellular machinery for successful infection, including viral genome replication, protein translation, and intracellular trafficking [7]. Previous studies have identified several host factors involved in TuMV infection, such as eukaryotic translation initiation factors (eIFs) and membrane trafficking components [8,9].

Chloroplasts serve as a crucial hub for integrating environmental stimuli and determining downstream responses in plants, extending their role beyond photosynthesis [10]. Extensive research has demonstrated that chloroplasts play a significant role in plant immunity, with multiple pathogenic factors associated with chloroplast immunity identified in viruses, bacteria, fungi, and oomycetes [11,12,13]. Many host factors related to chloroplast were reported to be involved in viral infection, such as chloroplast phosphoglycerate kinase (chl-PGK), photosystem II oxygen evolution complex protein 1 (PsbO1), rubisco small subunit (RbCS), the large subunit ribosomal protein 1 (NbRPL1), the photosystem I (PSI) subunit (PSaC), and ATP synthase subunit α (ATPsyn-α) [14,15,16,17,18].

In higher organisms, chloroplast proteins are partially encoded by the chloroplast genome and translated by prokaryotic-type 70S ribosomes within chloroplasts [19]. The 70S ribosomes consist of a 50S large subunit composed of 31 proteins homologous to those in *Escherichia coli*, with nine encoded by the plastid genome and 22 by the nuclear genome, and a 30S small subunit composed of 21 proteins, with 12 encoded by the plastid genome and nine by the nuclear genome [19]. Additionally, plastid ribosomes contain plastid-specific ribosomal proteins (PSRPs), which are not homologous to those found in *E. coli*. Initially identified in spinach (*Spinacia oleracea*), PSRPs are widely present in other higher plants and are encoded by nuclear genes, including four 30S subunit proteins (PSRP1-4) and two 50S subunit proteins (PSRP5-6) [20]. Subsequent studies revealed that PSRP1 is not a specific ribosomal protein but a plastid-specific ribosome-associated translation factor homologous to the *E. coli* cold shock protein pY [21]. The molecular functions of PSRPs are relatively understudied; in Arabidopsis, *PSRP3*, *PSRP4*, and *PSRP5* are essential for normal plant growth and development, while *PSRP2* and *PSRP6* knockdown mutations do not affect plant growth, and their functions remain unknown [22]

In this study, we confirmed interactions between TuMV and BcPSRP2 and BcPSRP4, motivated by their emerging roles in viral infections [23] and their unique properties and conservation across plant species [22]. To conduct a comprehensive analysis, we identified and cloned all *BcPSRPs* in non-heading Chinese cabbage (*Brassica campestris* ssp. *chinensis*), resulting in the isolation of seven *BcPSRPs*. Further interaction assays demonstrated that the VPg protein (virus protein, genome-linked) of TuMV interacts with BcPSRP2/4 proteins and alters their subcellular localization. BcPSRP2/4 can partially co-localize with TuMV viral replication complexes. Overexpressing *BcPSRP2/4* can enhance the cell-to-cell movement of TuMV. In Arabidopsis plants with a *psrp2* mutation, downregulation of *PSRP2* was found to inhibit TuMV infection, indicating that *PSRP2* facilitates viral infection. Finally, we found that silencing *BcPSRP2/4* using virus-induced gene silencing (VIGS) resulted in milder symptoms upon TuMV infection in non-heading Chinese cabbage. This research not only elucidates the molecular functions of *PSRPs* but also suggests that, given *PSRP2* is not essential for plant growth and development, mutations in *BcPSRP2* could potentially be applied to breeding Chinese cabbage with enhanced tolerance to TuMV, offering a sustainable strategy for the cabbage industry.

## 2. Results

### 2.1. The Identification of BcPSRPs in Non-Heading Chinese Cabbage

To investigate the molecular mechanisms of TuMV interaction with its host, we focused on *PSRPs* due to their emerging roles in viral infections [23]. Based on prior evidence of the unique properties and conservation across plant species [22], we validated the interaction of PSRP2 and its homolog, PSRP4, with TuMV components. This prompted a systematic characterization of their orthologs in non-heading Chinese cabbage (*Brassica campestris* ssp. *chinensis*) to elucidate their roles during infection.

**Figure 1:**
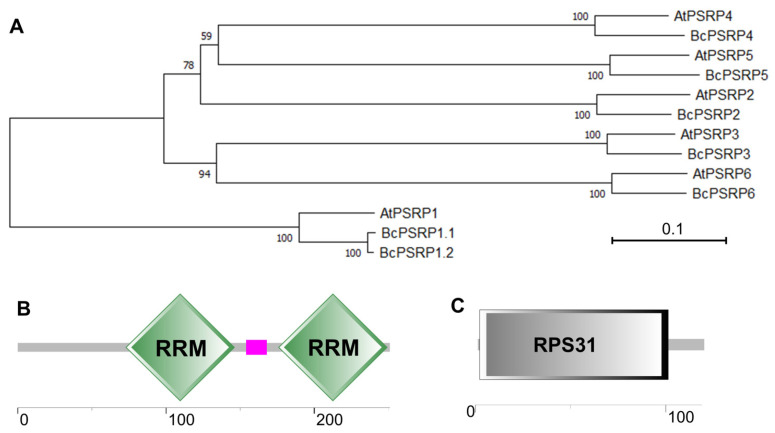
Evolutionary analysis and domain protection of the proteins of non-heading Chinese cabbage and Arabidopsis PSRPs. (**A**) The evolutionary relationships among plastid-specific ribosomal proteins (PSRPs) were reconstructed using maximum likelihood (ML) methodology. The analysis included all six Arabidopsis thaliana PSRP homologs (AtPSRP1-6) and seven non-heading Chinese cabbage orthologs (BcPSRP1.1/1.2/2/3/4/5/6). Numeric values at nodes indicate bootstrap support percentages, reflecting the robustness of each clade. (**B**,**C**) illustrate the protein domain architectures of BcPSRP2 and BcPSRP4, with a scale bar indicating length in amino acid residues. RRM, RNA recognition motif; RPS31, Ubi3 precursor, which is part of mature 60S and 40S ribosomal subunits.

Through BLAST v2.10.0+ homology searches against the non-heading Chinese cabbage protein database using Arabidopsis PSRPs (AtPSRP1-6), seven BcPSRPs were identified: BcPSRP1.1, BcPSRP1.2, BcPSRP2, BcPSRP3, BcPSRP4, BcPSRP5, and BcPSRP6. Using cDNA from the non-heading Chinese cabbage cultivar ‘49Caixin’ as a template, all BcPSRPs were successfully cloned. Sequence alignment revealed high conservation among homologous proteins from Arabidopsis, though PSRP1 exhibited greater divergence compared to other PSRPs. Specifically, PSRP2-PSRP6 each showed one-to-one correspondence between non-heading Chinese cabbage and Arabidopsis proteins; PSRP1 displayed two non-heading Chinese cabbage proteins corresponding to one Arabidopsis homolog (Figure 1A). The structural conservation patterns suggest evolutionary functional conservation among PSRP family members, with PSRP1 potentially undergoing distinct evolutionary trajectories in non-heading Chinese cabbage.

To delve deeper into BcPSRP2, we undertook a structural domain prediction, revealing that it harbors two RNA recognition motifs (RRMs) (Figure 1B). These motifs are commonly found in RNA-binding proteins and play crucial roles in RNA-protein interactions [24]. In contrast, BcPSRP4 features a typical RPS31 domain, which is integral to both the 60S and 40S ribosomal subunits (Figure 1C). The process of linear ubiquitination followed by cleavage of RPS31 is essential for the efficient assembly and functional integrity of the 40S ribosomal subunit [25].

### 2.2. BcPSRP2/4 Were Localized to Chloroplasts

**Figure 2:**
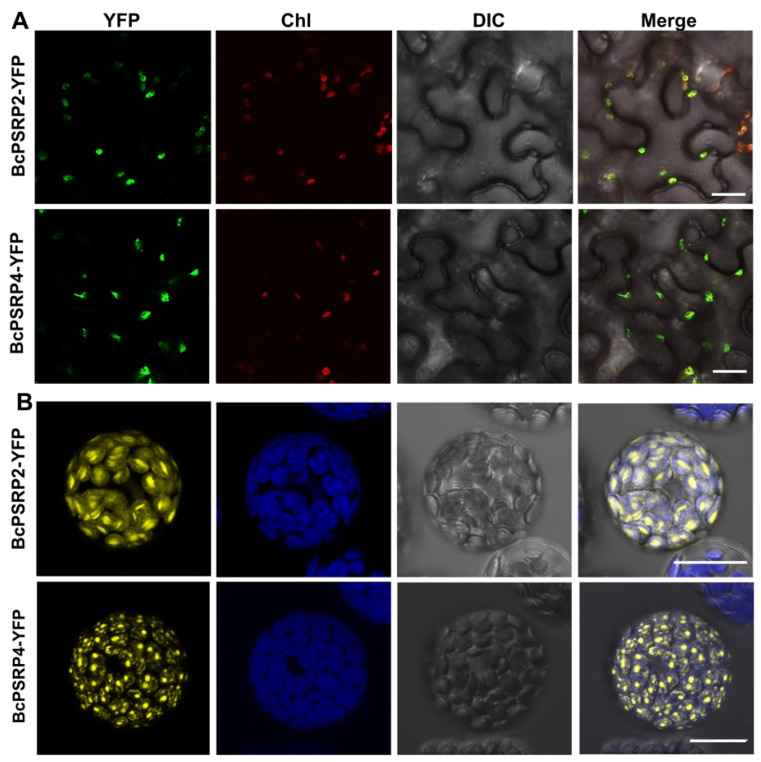
BcPSRP2/4 were localized to chloroplasts. (**A**) Confocal laser scanning microscopy images of YFP-tagged proteins expressed in *Nicotiana benthamiana* leaf epidermal cells via *A. tumefaciens*-mediated transient transformation (strain GV3101). Images were acquired at 48 h post-infiltration. (**B**) Fluorescence microscopy analysis of YFP-fusion proteins expressed in non-heading Chinese cabbage protoplasts via polyethylene glycol (PEG)-mediated transformation. YFP fluorescence: excitation 514 nm/emission 527 nm (yellow) Chlorophyll autofluorescence: excitation 543 nm/emission 650–750 nm (red or blue). Chl, chloroplast; YFP, yellow fluorescent protein; DIC, differential interference contrast microscopy. Scale bars = 20 μm.

Confocal microscopy analysis demonstrated that both BcPSRP2 and BcPSRP4 are chloroplast-targeted proteins when expressed as YFP fusions in tobacco leaves and non-heading Chinese cabbage protoplasts (Figure 2A,B). While sharing this organellar destination, the two proteins appeared to show subtle differences in their distribution patterns within chloroplasts: BcPSRP2 displayed a more uniform stromal localization, whereas BcPSRP4 formed some punctate structures (Figure 2B). This differential suborganellar targeting suggests functional specialization, despite their typical plastid localization—a phenomenon well-documented for other nuclear-encoded chloroplast proteins involved in RNA processing and photosynthetic complex assembly [26].

The punctate aggregation pattern of BcPSRP4 may reflect its association with specific chloroplast substructures, such as plastoglobules, transcriptionally active chromosomes (TACs), or RNA processing bodies—all of which are known to form viral replication complexes during plant virus infections [17]. Particularly intriguing is the potential link between these puncta and TuMV infection, as chloroplast membranes frequently serve as platforms for the assembly of the potyviral replication complex [27]. The subtle differences in localization patterns between BcPSRP2 and BcPSRP4 may suggest potential differences in their roles during viral pathogenesis.

### 2.3. BcPSRP2/4 Interacts with TuMV VPg and Enters the Nucleus upon Viral Infection

**Figure 3:**
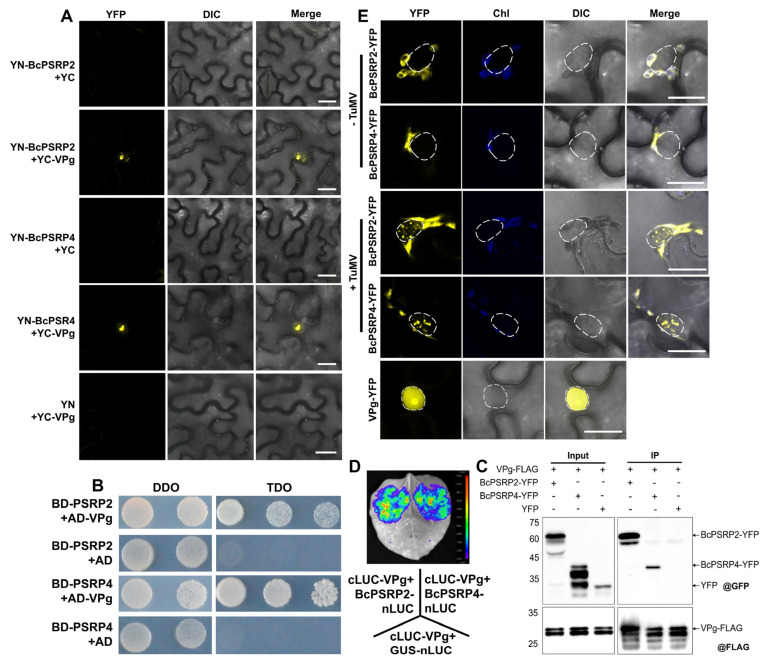
BcPSRP2/4 interacts with TuMV VPg and enters the nucleus upon viral infection. (**A**) Bimolecular fluorescence complementation (BiFC) assay was used to confirm the interaction between BcPSRP2/4 and TuMV VPg protein. Empty vectors of YN or YC serve as the negative controls. (**B**) A yeast two-hybrid (Y2H) assay was used to confirm the interaction between BcPSRP2/4 and TuMV VPg protein, as evidenced by yeast growth on high-stringency TDO medium, indicating specific interactions between BcPSRP2/4 (pGBKT7) and VPg (pGADT7). Empty vectors (pGBKT7 and pGADT7) served as negative controls. (**C**) Co-Immunoprecipitation (Co-IP) assay was used to confirm the interaction between BcPSRP2/4 and TuMV VPg protein in vivo, showing BcPSRP2/4-YFP co-precipitated with VPg-FLAG using anti-FLAG M2 affinity gel, detected by anti-GFP and anti-FLAG antibodies. The YFP empty vector was used as a negative control. (**D**) Interaction between the C-terminal half of luciferase-VPg (cLUC-VPg) and the BcPSPR2/4 N-terminal half of luciferase (BcPSPR2/4-nLUC) by luciferase complementation assay (LCA). GUS-nLUC was used as a control. The color gradient on the right ranges from blue (background) to red (maximum intensity), representing photon flux values from 1000 to 5500 photons/s/cm^2^/sr. (**E**) Tobacco leaves were co-infiltrated with TuMV and BcPSRP2/4-YFP fusion constructs. Fluorescence microscopy was used to visualize the distribution of YFP-tagged proteins. No-TuMV-infected plants served as the negative control. VPg-YFP is used to demonstrate nuclear localization characteristics. Dashed lines demarcate nuclei. DDO, double dropout supplements; TDO, triple dropout supplements; IP, immunoprecipitation; YFP, yellow fluorescent protein; Chl, chloroplast; DIC, differential interference contrast. Scale bars = 20 μm.

To elucidate the viral proteins that interact with BcPSRP2/4, we employed a bimolecular fluorescence complementation (BiFC) assay to screen 11 TuMV proteins: P1, HC-Pro, P3, P3N-PIPO, 6K1, CI, 6K2, VPg, NIa, NIb, and CP. The result revealed that BcPSRP2/4 only interacted with the viral protein VPg. VPg functions by mimicking the 5′ cap structure of mRNA, covalently attaching to the 5′ terminus of the TuMV genome, thereby facilitating the initiation of viral translation [28]. Importantly, VPg exhibits specific nuclear localization, accumulating in the nucleolus (Figure 3A). These interactions were further confirmed using yeast two-hybrid (Y2H) and co-immunoprecipitation (Co-IP) assays (Figure 3B,C). In the Y2H assay, co-expression of BcPSRP2/4 (pGBKT7) and VPg (pGADT7) supported yeast growth on high-stringency SD/-Leu/-Trp/-His medium, confirming specific interactions, while empty vectors showed no growth (Figure 3B). Similarly, the Co-IP assay in *Nicotiana benthamiana* leaves demonstrated that BcPSRP2/4-YFP was specifically co-precipitated with VPg-FLAG, as detected by anti-GFP and anti-FLAG antibodies, with no interaction observed with the YFP empty vector control (Figure 3C). A Luciferase complementation assay (LCA) was also employed to verify this interaction, using the C-terminal half of luciferase fused to VPg (cLUC-VPg) and the N-terminal half of luciferase fused to BcPSPR2/4 (BcPSPR2/4-nLUC). The GUS-nLUC was used as a negative control. The result revealed distinct interaction patterns between viral and host proteins. Strong luminescent signals were detected in leaves co-expressing cLUC-VPg with BcPSRP2/4-nLUC (Figure 3D), indicating a specific interaction between VPg and BcPSRP2/4 proteins. In contrast, the cLUC-VPg/GUS-nLUC control combination showed negligible luminescence signals (Figure 3D).

Additionally, BiFC experiments demonstrated that VPg alters the subcellular localization of BcPSRP2/4, redirecting them from chloroplasts to the nucleus (Figure 3A). In plants infected with TuMV, transient expression of BcPSRP4, followed by fluorescence observation 48 h later, showed that a portion of BcPSRP4 entered the nucleus and formed aggregates (Figure 3E). This suggests that VPg plays a role in modulating the localization and aggregation of BcPSRP2/4 within the nucleus, potentially impacting their functions during TuMV infection.

### 2.4. BcPSRP2/4 Partially Co-Localized with the TuMV Viral Replication Complex

**Figure 4:**
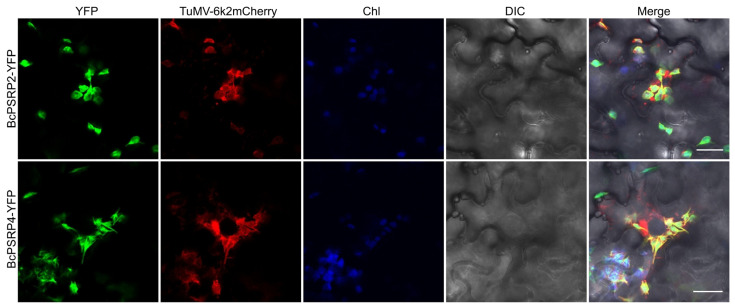
Partial co-localization of BcPSRP2/4 with TuMV viral replication complexes. Co-expression of TuMV::6K2mCherry infectious clone with BcPSRP2/4-YFP in *N. benthamiana* leaves was conducted. The 6K2mCherry fusion protein labels the viral replication complex, allowing visualization of its localization. Fluorescence imaging was used to assess co-localization of BcPSRP2/4-YFP with the 6K2mCherry-labeled viral replication complex. YFP, yellow fluorescent protein; Chl, chloroplast; DIC, differential interference contrast. Scale bars = 20 μm.

After a 72 h incubation, we used confocal laser scanning microscopy to examine the localization of TuMV::6K2-mCherry and BcPSRP2/4-YFP in tobacco leaves. The results, as depicted in Figure 4, indicate that BcPSRP2/4 partially co-localizes with the 6K2-mCherry protein, which serves as a marker for the viral replication complex. This co-localization suggests that BcPSRP2/4 can be recruited by the viral replication complex, potentially participating in the replication process of TuMV.

### 2.5. Overexpression of PSRP2/4 Promotes TuMV Intercellular Movement

**Figure 5:**
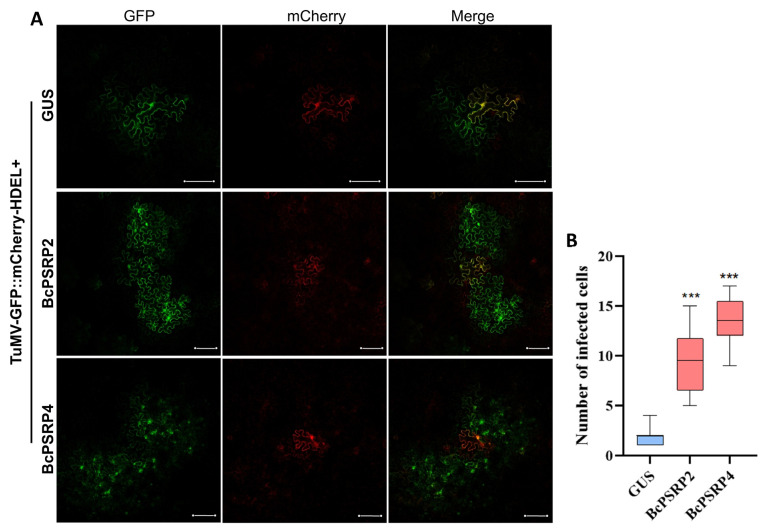
Overexpression of PSRP2/4 promotes TuMV intercellular movement. (**A**) The infectious clone TuMV-GFP//mCherry-HDEL was co-expressed with BcPSRP2/4, using GUS as a negative control. GFP labels newly produced viral particles after they move, while mCherry marks the initially inoculated cells. Scale bars = 20 μm. (**B**) Statistical analysis of mobile cell numbers. *** indicates *p* < 0.0001.

Here, we used TuMV-GFP//mCherry-HDEL to examine the movement of the virus. It is designed as a dual-expression system to facilitate visualization of viral and cellular components in *N. benthamiana* leaf cells [29]. This construct contains two distinct expression cassettes driven by the CaMV 35S promoter: one drives the production of mCherry fused to an endoplasmic reticulum (ER) retention signal (mCherry-HDEL), localizing red fluorescence to the ER lumen, and the other encodes the TuMV genome tagged with green fluorescent protein (GFP). Upon agroinfiltration into leaf cells, the 35S promoter enables initial transcription of both cassettes in primary infected cells, producing authentic viral RNA from the TuMV-GFP cassette that replicates and spreads via progeny virions lacking the promoter sequence. This results in distinct green fluorescence from the TuMV-GFP and red fluorescence from mCherry-HDEL, allowing clear visualization of their respective localizations. As shown in Figure 5A,B, co-expression of TuMV-GFP//mCherry-HDEL with BcPSRP2/4 significantly enhanced viral cell-to-cell movement compared to the GUS-negative control (*p* < 0.0001). Quantitative analysis revealed that GFP-tagged viral particles exhibited an expanded distribution pattern in BcPSRP2/4-overexpressing cells, whereas the movement of GFP-tagged viral particles was highly restricted in GUS-expressing controls (Figure 5A). This distinct movement phenotype suggests that BcPSRP2/4 may either facilitate the viral movement machinery or modulate plasmodesmal permeability to promote intercellular trafficking, as reported in a recent study [30].

### 2.6. Down-Regulation of PSRP2 Is Associated with Milder Disease Symptoms in Arabidopsis

**Figure 6:**
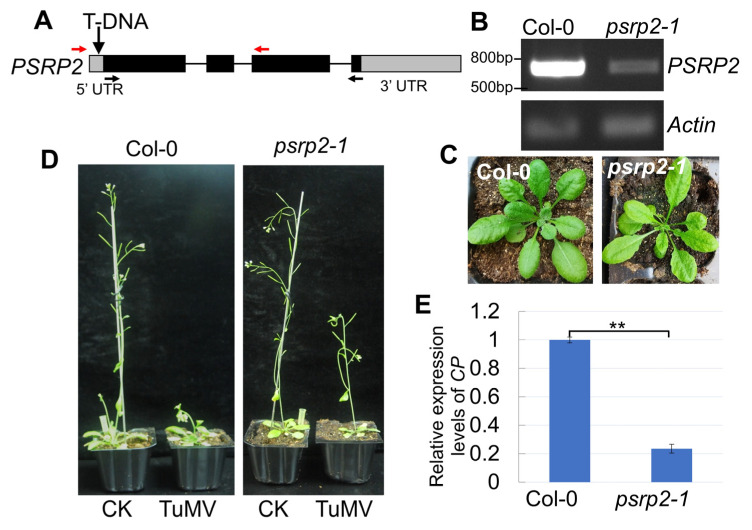
Down-regulation of *PSRP2* is associated with milder disease symptoms in Arabidopsis. (**A**) Schematic representation of the T-DNA insertion site in the Arabidopsis *psrp2-1* mutant line, showing disruption of the *AtPSRP2* 5′UTR region. Red arrows indicate genotyping primers LP/RP flanking the T-DNA insertion site. Black arrows denote RT-PCR primers F/R for *AtPSRP2* expression quantification. (**B**) RT-PCR analysis confirms a significant reduction in the AtPSRP2 transcript level in the *psrp2-1* mutant compared to wild-type Col-0 plants, using *Actin* as an internal control. (**C**) Comparative phenotypic analysis revealed no developmental abnormalities in *psrp2-1* mutants under normal growth conditions. (**D**) Typical viral symptom development following pathogen inoculation in *psrp2-1* mutants. CK represents the negative control without TuMV infection. (**E**) Viral challenge assay demonstrating milder symptoms upon TuMV infection in the *psrp2-1* mutant. Quantitative detection of viral coat protein (*CP*) gene accumulation by qRT-PCR (** *p* < 0.01), normalized to *Actin* expression.

To investigate the functions of the *PSRP* gene in cruciferous species lacking mutant resources (non-heading Chinese cabbage), we systematically screened Arabidopsis T-DNA insertion lines targeting both *AtPSRP2* and *AtPSRP4* homologs. Through systematic screening of multiple Arabidopsis T-DNA insertion lines targeting both PSRP homologs, we successfully isolated a *PSRP2* knockdown mutant (*psrp2-1*) but failed to identify viable *PSRP4* mutants despite extensive efforts. The *psrp2-1* mutant line harbors a T-DNA insertion in the 5′ untranslated region (5′ UTR) of *PSRP2*. This insertion significantly impairs *PSRP2* expression, as confirmed by RT-PCR analysis (Figure 6A,B). The absence of recoverable *PSRP4* mutants may reflect technical constraints associated with its compact genomic architecture- the relatively short coding sequence length (360 bp) substantially reduces the probability of obtaining viable insertion events without disrupting essential functional domains.

The *PSRP2* expression in this mutant was approximately one-tenth that of the wild-type Col-0 based on semi-quantitative reverse transcription PCR (RT-PCR) analysis (Figure 6B). Notably, the growth of *psrp2-1* mutants was comparable to that of Col-0, suggesting that *PSRP2* is not indispensable for plant growth (Figure 6C). Upon inoculation with TuMV, the *psrp2-1* mutants exhibited milder symptoms, such as reduced plant height, compared to virus-inoculated wild-type plants (Figure 6D). Furthermore, the expression of the TuMV coat protein (*CP*) gene was significantly lower in the mutants than in wild-type plants (Figure 6E). These findings suggest that *PSRP2* facilitates viral infection, and downregulating *PSRP2* results in milder symptoms upon TuMV infection without adversely affecting plant growth. This suggests a potential strategy for improving plant resistance to TuMV by modulating *PSRP2* expression.

### 2.7. Silencing BcPSRP2/4 Resulted in Milder Symptoms upon TuMV Infection in Brassica rapa

**Figure 7:**
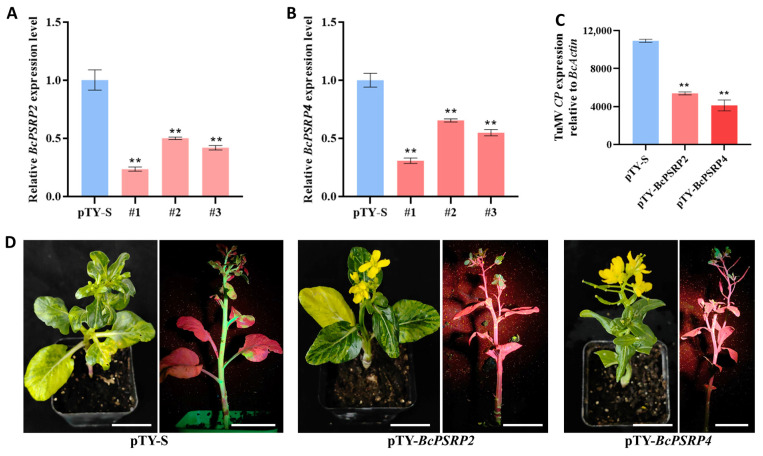
Silencing *BcPSRP2/4* causes milder symptoms to TuMV in non-heading Chinese cabbage. (**A**,**B**) Expression analysis of *BcPSRP2/4* in silenced ‘49Caixin’. (**C**) TuMV-CP expression levels after silencing *BcPSRP2* and *BcPSRP4* in ‘49Caixin’. (**D**) Phenotypes of TuMV-GFP infection in *BcPSRP2*- and *BcPSRP4*-silenced ‘49Caixin’. The left panel displays disease symptoms, while the right panel shows the distribution of TuMV-GFP under UV light. Scale bar = 2 cm. ** represents *p* < 0.01.

To functionally characterize *BcPSRP2/4* in non-heading Chinese cabbage, we employed virus-induced gene silencing (VIGS) using the pTY-*BcPSRP2/4* construct in ‘49Caixin’ cultivar, with pTY-S vector serving as an empty control. Quantitative reverse transcription PCR (qRT-PCR) analysis revealed efficient silencing efficacy, showing significant downregulation (>70% reduction) of *BcPSRP2/4* transcripts in both independent VIGS lines compared to controls at 14 days post-infiltration (Figure 7A,B).

To evaluate the antiviral effects of *BcPSRP2/4* silencing, systemic leaves from successfully silenced plants were challenge-inoculated with TuMV-GFP. Three key findings emerged: molecular analysis demonstrated that *BcPSRP2/4*-silenced plants exhibited significantly reduced accumulation of viral *CP* transcripts (~60% decrease by qPCR; Figure 7C); Phenotypic observations revealed delayed symptom onset and reduced severity in silenced plants compared to controls developing typical mosaic/mottling symptoms; Fluorescence imaging under UV light revealed substantially smaller GFP-expressing areas in silenced plants (Figure 7D), indicating impaired viral spread.

*BcPSRP2/4* suppression correlates with reduced relative virus titer, delayed symptom onset, and impaired TuMV spread, suggesting it acts as a susceptibility gene. This may occur by facilitating the assembly of the viral replication complex, compromising host RNA interference pathways, or modifying the efficiency of plasmodesmal trafficking. These findings suggest that *BcPSRP2/4* could serve as a potential target for developing Brassica crops with reduced symptom severity caused by TuMV, potentially through CRISPR-mediated gene editing or RNAi-based approaches.

## 3. Discussion

Our study provides compelling evidence that TuMV VPg interacts with PSRP2 and PSRP4, revealing novel host factors that facilitate viral infection. The interaction between TuMV VPg and BcPSRP2/4 proteins, coupled with their altered subcellular localization during infection, suggests that TuMV may co-opt these ribosomal proteins to support viral replication and cell-to-cell movement. This is further supported by the partial co-localization of BcPSRP2/4 with TuMV replication complexes, implying their potential role in viral RNA translation or replication complex assembly. The functional significance of *PSRP2* in TuMV infection was clearly demonstrated by our findings, which showed that Arabidopsis psrp2 mutants exhibited milder symptoms in response to TuMV infection. At the same time, overexpression of *BcPSRP2/4* enhanced viral cell-to-cell movement.

Regarding PSRP2’s role as a proviral factor in TuMV infection, we hypothesize that it supports viral protein synthesis or indirectly stabilizes viral RNA through its chloroplast functions. More specifically: (1) PSRP2 may modulate chloroplast ribosomal activity to enhance the production of host resources (e.g., amino acids or energy metabolites) that indirectly bolster cytoplasmic viral translation; (2) it could stabilize viral RNA by influencing RNA-binding interactions or complexes near chloroplast membranes, where TuMV’s 6K2-mediated replication complexes have been reported to localize [27,31]; or (3) PSRP2 might alter chloroplast membrane dynamics or stress responses, creating a permissive environment for viral vesicle docking and replication without direct viral entry into the organelle. The observation that silencing *BcPSRP2/4* led to milder symptoms of TuMV infection in Chinese cabbage further strengthens this conclusion and highlights the potential of targeting these non-essential ribosomal proteins for antiviral strategies.

In studies related to plant viruses, more than ten host proteins localized to chloroplasts have been found to enhance viral pathogenicity by facilitating chloroplast targeting, replication, and movement [12,17,32]. For instance, chloroplast phosphoglycerate kinase (chl-PGK) assists in the localization of bamboo mosaic virus (BaMV, *Potexvirus bambusae*) RNA to chloroplasts, and silencing *chl-PGK* reduces the accumulation of BaMV coat protein in plants [14]. This suggests that chloroplast-targeted metabolic enzymes, such as chl-PGK, may facilitate viral RNA trafficking—a function potentially shared by plastid ribosomal proteins, such as PSRPs, given their RNA-binding properties. Similarly, the 6K2 protein of tobacco vein banding mosaic virus (TVBMV, *Potyvirus nicotianavenaobscurum*) interacts with photosystem II oxygen evolution complex protein 1 (PsbO1) in tobacco, thereby regulating viral replication [15]. Such findings suggest that other chloroplast-localized proteins, including PSRPs, could also influence viral replication complexes through membrane association domains.

The well-documented interactions between viral movement proteins and key chloroplast components reveal conserved targeting strategies. For example, tomato mosaic tobamovirus (ToMV, *Tobamovirus tomatotessellati*) MP binds to the rubisco small subunit, while abutilon mosaic virus (AbMV, *Begomovirus bauri*) MP recruits cpHSC70-1 chaperones during intercellular transport [33,34,35]. These examples parallel how PSRPs might serve as adaptors between viral cargos and host transport machinery due to their dual localization patterns. Recent reports have also shown that chloroplast ribosomal proteins are involved in regulating viral infections. For example, the large subunit ribosomal protein 1 (NbRPL1) of tobacco chloroplasts interacts with the NIb protein of TVBMV, competing with NbBeclin1 for NIb binding and reducing NbBeclin1-mediated NIb degradation; silencing *NbRPL1* decreases viral replication [36]. Most recently, Qin et al. demonstrated that potyviral HCPro universally hijacks NbRbCS, forming conserved movement complexes at plasmodesmata [16]. This reinforces how photosynthetic components are repurposed as molecular scaffolds—a strategy likely extending beyond RbCS, given our current understanding of other stromal proteins, like PSRPs, participating in similar processes during infection. Collectively, these studies establish a paradigm in which diverse chloroplast proteins are co-opted through distinct yet complementary mechanisms during viral pathogenesis. The emerging pattern suggests an evolutionary convergence, where photosynthetic components, including ribosomal proteins, metabolic enzymes, and reaction center subunits, are preferentially targeted by multiple virus families—potentially including the yet uncharacterized roles of PSRPs in these processes.

Our findings on the hijacking of PSRPs by TuMV have further illuminated the emerging paradigm of chloroplast-virus interplay. While previous studies have established that chloroplast-targeted proteins, such as RbCS and cpHSC70-1, facilitate viral movement through direct interaction with viral movement proteins (MPs) [33,34], we demonstrate that PSRPs—core components of chloroplast ribosomes—orchestrate viral pathogenesis through a dual regulatory axis. First, BiFC, H2Y, Co-IP, and LCAs revealed direct interaction between TuMV VPg and BcPSRP2/4 (Figure 3A–D), with confocal microscopy showing VPg-induced re-localization of PSRP2/4 from chloroplasts to viral replication complexes (VRCs) at the chloroplast-cytoplasmic interface (Figure 4). This redistribution mirrors the NbRbCS-mediated movement complex assembly [16], suggesting convergent exploitation of chloroplast-nucleus coordinated proteins by potyvirids. Functional studies unveiled PSRPs’ dual roles in viral propagation: (i) *PSRP2/4* overexpression enhanced TuMV cell-to-cell movement by 2.3-fold (Figure 5), likely through PSRP2/4’s RNA-binding domains facilitating VRC anchoring to chloroplast envelopes for efficient RNA shuttling; (ii) *psrp2* mutants exhibited 67% reduction in viral accumulation (Figure 6), which starves viral complexes of RbCS—a key scaffold in potyviral movement [16]. Crucially, VIGS-mediated silencing of *BcPSRP2/4* in Chinese cabbage conferred broad-spectrum resistance without compromising growth (Figure 7), underscoring its non-essential role in development—a strategic advantage over eIF4E-targeted resistance, which often impairs host fitness [7].

Our results demonstrate that TuMV has evolved a sophisticated strategy to hijack host PSRP through specific interaction with VPg, leading to its nuclear re-localization during infection (Figure 3E). The formation of phase-separated nuclear aggregates containing both viral and host components suggests that these structures may serve as specialized microenvironments, facilitating viral replication while potentially disrupting normal host RNA metabolism. The recruitment of BcPSRP2/4 to VRCs underscores the evolutionary arms race between viruses and hosts: while TuMV exploits host machinery for replication, plants may deploy proteins like BcPSRP2/4 to restrict viral propagation. Similar mechanisms are observed in other potyviruses, where host RNA-binding proteins modulate viral RNA synthesis [9,37]. This work reveals a novel mechanism by which potyviruses spatially reprogram host factors through direct protein–protein interactions, thereby expanding our understanding of how viruses manipulate cellular trafficking pathways. These findings not only provide new insights into plant-virus interactions but also identify potential targets for developing antiviral strategies that aim to disrupt these critical host–pathogen interfaces.

In summary, BcPSRP2 and BcPSRP4 interact directly with TuMV VPg, thereby disrupting its localization and promoting viral cell-to-cell spread. Overexpression enhances infection, while Arabidopsis *psrp2* knockdown results in milder symptoms upon TuMV infection, confirming its proviral function. Notably, VIGS suppression of *BcPSRP2/4* also caused milder symptoms without growth costs. Our findings carry transformative implications for sustainable agriculture. Unlike conventional resistance genes that may impose trade-offs between defense and yield, *PSRP2* knockdown exhibited no biomass penalty under greenhouse conditions. This aligns with the chloroplast’s metabolic buffering capacity, where residual PSRP4 compensates for PSRP2 loss in translation while maintaining sufficient rubisco activity. Leveraging CRISPR-Cas9 to engineer *PSRP2* allelic variants (e.g., truncations preserving ribosome binding but lacking VPg interaction domains) may offer a pathway toward Brassica cultivars with alleviated symptom severity to TuMV infection—a strategy under preliminary testing in our ongoing trials with Brassica hybrids.

## 4. Materials and Methods

### 4.1. Plant Cultivation and Viral Inoculation

The non-heading Chinese cabbage cultivar ‘49Caixin’ and *N. benthamiana* were grown in containers filled with a 3:1 mixture of peat moss and perlite. The plants were maintained at a constant temperature of 25 °C under a photoperiod of 16 h of light and 8 h of darkness.

*A. thaliana* seeds were surface-sterilized with 75% (*v*/*v*) ethanol for 10 min, followed by two washes with 100% ethanol, and then dried on a sterile bench. Subsequently, the seeds were placed on half-strength Murashige and Skoog (MS) agar plates and stratified in a refrigerator for three days. Ultimately, the Arabidopsis seeds germinated, and the resulting seedlings were grown in a controlled environment within a tissue culture chamber, with a photoperiod of 16 h of light and 8 h of darkness at a temperature of 22 °C.

Viral inoculation was conducted using symptomatic TuMV-infected ‘49Caixin’ leaves as the source of infection, as described in previous research [38]. The inoculum was prepared by homogenizing leaf tissue in Phosphate-Buffered Saline (PBS) at a 1:10 (*w*/*v*) ratio, with the addition of carborundum abrasive during grinding to facilitate viral release. Before inoculation, leaves were dusted with carborundum to create microabrasions, followed by application of 100 µL inoculum per plant. Control plants were mock-inoculated with PBS buffer alone. After 20 min of incubation, leaves were rinsed thoroughly with distilled-deionized water (ddH_2_O) to remove residual particles.

### 4.2. Genome-Wide Characterization of the BcPSRPs

The non-heading Chinese cabbage (NHCC001) protein database [39] was searched using BLASTP v2.10.0+ (e-value < 1 × 10^−50^) with Arabidopsis PSRPs [22] as the query. The selected BcPSRPs were evolutionarily analyzed by clustering using MEGAX [40] and the maximum likelihood method. Protein structure domain was forecasted using SMART (https://smart.embl.de//).

### 4.3. Subcellular Localization and Bimolecular Fluorescence Complementation (BiFC)

The coding sequence (CDS) of *BcPSRP2/4* was cloned in-frame with GFP using Gateway recombination. *A. tumefaciens* strain GV3101 harboring either the BcPSRP2/4-YFP fusion construct or YFP-only control vector was resuspended in infiltration buffer (optical density at 600 nm [OD600] = 0.5). Transient transformation assays were performed on fully expanded leaves of 4-week-old *N. benthamiana* plants through Agrobacterium-mediated leaf infiltration.

The optimized transient expression protocol, based on previous research [29] in ‘49Caixin’ mesophyll protoplasts, involves the following steps: Fresh leaf segments (approximately 3 cm^2^) were immobilized with adhesive tape, followed by gentle removal of the lower epidermis using a tape-sandwich technique. The peeled tissues were subjected to enzymatic digestion in pre-activated solution (55 °C, 10 min) for 1–3 h at room temperature. Protoplasts were subsequently washed twice with ice-cold W5 buffer (154 mM NaCl, 125 mM CaCL_2_, 5 mM KCl, 5 mM Glucose, 2 mM MES, pH5.7) for 600× *g*, 1 min, incubated on ice for 30 min, and adjusted to 2–5 × 10^5^ cell/mL using MMg buffer (0.4 M Mannitol, 15 mM MgCl_2_, 4 mM MES, pH5.7). Transformation was achieved by combining 600 μL protoplast suspension with 20–30 μg BcPSRP2/4-YFP plasmid DNA and 600 μL PEG4000 solution (40% *w*/*v* PEG4000, 0.1 M CaCl_2_, 0.2 M D-Mannitol, pH 5.7), followed by 15–20 min room temperature incubation. After three rounds of W5 washing, the transfected protoplasts were cultured for 40 h before undergoing confocal imaging or RNA extraction.

The BiFC system was employed to investigate Protein–Protein Interactions using vectors pEarleyGate201-YC and pEarleyGate202-YN [40]. The correctly sequenced expression vectors were introduced into *A. tumefaciens* strain GV3101. To examine interactions, YN-tagged BcPSRP2 and BcPSRP4 were co-injected with YC-tagged proteins (VPg or GUS) in equal proportions, maintaining a final OD600 of 0.3 for each component. All the fluorescence signals were analyzed 48 h post-infiltration using a Leica SP8 STED 3X confocal microscope (Leica Microsystems, Wetzlar, Germany) equipped with 488 nm excitation and 500–550 nm emission filters.

### 4.4. Cell-to-Cell Movement Assay

The cell-to-cell movement assay was performed following established methodologies with modifications [29]. Briefly, recombinant *A. tumefaciens* strains carrying TuMV-GFP//mCherry-HDEL, as described in [29], constructs were co-infiltrated with BcPSRP2/4-FLAG expressing bacteria (final OD600 = 0.5) into fully expanded leaves of *N. benthamiana* plants at the 4-week growth stage. The working concentration for viral constructs was adjusted to OD600 = 0.0001 in infiltration buffer (10 mM MES, 10 mM MgCl_2_, 150 μM acetosyringone). At 48 h post-infiltration, infection foci were visualized using a Leica TCS SP8 confocal microscope (Leica Microsystems, Wetzlar, Germany) with excitation/emission wavelengths of 488/500–550 nm for GFP and 561/570–620 nm for mCherry. To quantify viral cell-to-cell movement, infection foci containing a single mCherry-HDEL primary infected cell, identified by red fluorescence, were selected. Adjacent GFP-positive cells surrounding each qualifying focus were manually counted to measure secondary spread events. A minimum of eight foci per sample were analyzed, and data were expressed as mean ± SD (*p* < 0.05, Student’s *t*-test).

### 4.5. Yeast Two-Hybrid (Y2H) Assay

To investigate Protein–Protein Interactions, the full-length coding sequences (CDS) of BcPSRP2 and BcPSRP4 were cloned into the pGBKT7 yeast two-hybrid vector. Constructs were co-transformed into strain Y2HGold using the lithium acetate method. Transformants were selected on Synthetic Dropout (SD) medium lacking leucine and tryptophan (SD/-Leu/-Trp). Interactions were assessed by growth on high-stringency SD medium lacking leucine, tryptophan, and histidine (SD/-Leu/-Trp/-His). Interaction analyses were conducted by monitoring yeast growth on selective media deficient in specific nutrients.

### 4.6. Co-Immunoprecipitation (Co-IP) Assay

To investigate the interaction between BcPSRP2/4 and VPg proteins, we employed a co-immunoprecipitation (Co-IP) assay in *N. benthamiana* leaves with some modifications [41]. Constructs of BcPSRP2/4-YFP and VPg-FLAG were transformed into *Agrobacterium tumefaciens* strain GV3101 and cultured to an OD600 of 0.5. Equal volumes of bacterial suspensions were mixed and co-infiltrated into 4-week-old *N. benthamiana* leaves. After 48 h, leaf samples (1 g per sample) were harvested, ground in liquid nitrogen, and extracted in lysis buffer. Cleared lysates were incubated with anti-FLAG M2 affinity gel (Sigma-Aldrich, St. Louis, MO, USA) at 4 °C for 2 h. Immunoprecipitated proteins were eluted by boiling in 2× SDS loading buffer for 10 min, separated by SDS-PAGE, and analyzed by immunoblotting using anti-FLAG M2 (1:10,000, Sigma-Aldrich) and anti-GFP (1:3,000, Yeasen Biotech, Shanghai, China) antibodies. A YFP empty vector was used as a negative control to confirm the specificity of the interaction.

### 4.7. Luciferase Complementation Assay (LCA)

The interaction between the C-terminal half of luciferase fused to VPg (cLUC-VPg) and the N-terminal half of luciferase fused to BcPSPR2/4 (BcPSPR2/4/GUS-nLUC) was assessed using LCA following an established protocol with modifications [42]. Briefly, recombinant *A. tumefaciens* strains GV3101 harboring cLUC-VPg and BcPSPR2/4-nLUC vectors were cultured to OD600 = 0.5, and then mixed at a 1:1 ratio with the infiltration buffer mentioned above. The bacterial suspension was infiltrated into the leaves of *N. benthamiana* (4-week-old plants) using a needleless syringe. 48 h post-infiltration, LUC signals in tobacco leaves were visualized using an In Vivo imaging system (PlantView100, Guangzhou Boluteng Instrument Co., Ltd., Guangzhou, China) after spraying leaves with 1 mM D-luciferin (potassium salt, Yeasen, Shanghai, China) dissolved in 0.01% Triton X-100.

### 4.8. Genetic Identification of Mutant Line

To identify homozygous *psrp2-1* mutants (SALK_140803C), we performed PCR-based genotyping using gene-specific primers LP: 5′-AACCTTTATCTCACGCCCTTC-3′ and RP: 5′-CAACGAAGAATCATGGAGTGG-3′, along with T-DNA left border primer LBb1.3: 5′-ATTTTGCCGATTTCGGAAC-3′. Homozygous mutants were confirmed by both the absence of wild-type amplification product and detection of a characteristic T-DNA insertion band through agarose gel electrophoresis. To validate the reduction in PSRP2 transcript levels, semi-quantitative reverse transcription PCR (RT-PCR) analysis was conducted in mutant plants using AtPSRP2-specific primers (F: 5′-ATGGCGACTTTCCTAACAAATGTT-3′; R: 5′-AGCCTTATTCACCCGAATCTTCT-3′), with *Actin2* (F: 5′-GACCTTTAACTCTCCCGCTATG-3′, R: 5′-GAGACACACCATCACCAGAAT-3′) serving as an internal control for normalization. Total RNA was extracted from Arabidopsis leaves (100 mg) using TRIzol reagent (Invitrogen, Waltham, MA, USA), and cDNA was synthesized using PrimeScript RT Master Mix (Takara, Dalian, China). PCR products were separated by 1.5% agarose gel electrophoresis and visualized with ethidium bromide. The viral accumulation was quantified by qRT-PCR targeting the *CP* gene (F: 5′-AATCCTATACACGCCGGAGCAGAC-3′; R: 5′-CTCCGTCAGTTCGTAATCAGC-3′) after the challenge of TuMV.

### 4.9. Virus-Induced Gene Silencing (VIGS)

The functional analysis of *BcPSRP2* and *BcPSRP4* genes was conducted using an optimized virus-induced gene silencing (VIGS) protocol [43]. Target-specific 40-mer oligonucleotides corresponding to conserved regions of *BcPSRP2* (CTGAAGAAAAACCCGCTTCAGACCCTAACGCAGAGTCCTC) and *BcPSRP4* (CTAATCGGAGCTCCTCCGCGACTCACCGTCCCATCATCTT) were designed to generate an 80 bp inverted repeat structure through reverse complementation. Recombinant pTY-BcPSRP2/4 vectors were commercially synthesized (GenScript, Nanjing, China) and amplified in *E. coli*. Plasmid DNA was purified using a commercial extraction kit (Tiangen Biotech, Beijing, China) for subsequent transformations. Gold microparticles (1.0 μm) coated with recombinant plasmids were ballistically introduced into two-true-leaf-stage plants using a helium-driven gene delivery system (Bio-Rad PDS-1000/He, Hercules, CA, USA). The bombardment parameters were as follows: a target distance of 9 cm, a helium gas pressure of 8.96 MPa (1300 psi), and a vacuum pressure of 0.36 MPa (26 inHg). Each construct was tested on six biological replicates per experiment across three independent trials. Systemic symptom development was monitored for 14 days post-bombardment, after which leaves exhibiting characteristic viral-induced phenotypes were harvested for molecular validation. Quantitative reverse transcription PCR (qRT-PCR) was performed to assess transcript suppression efficiency relative to empty vector controls (pTY-S). Significantly down-regulated plants were inoculated as above with TuMV. After the plants developed typical TuMV symptoms, viral accumulation was quantified by qRT-PCR targeting the *CP* gene.

### 4.10. Statistical Analysis

All experimental procedures were independently replicated a minimum of three times, unless explicitly stated otherwise. Quantitative results are expressed as mean values ± standard deviation (SD), derived from a representative set of biological replicates. Statistical comparisons were performed using two-tailed Student’s *t*-test, with significance thresholds set at *p* < 0.05 (*), *p* < 0.01 (**), and *p* < 0.001 (***).

## Data Availability

The original contributions presented in this study are included in the article. Further inquiries can be directed to the corresponding author.
